# The Motivation of Media Users and China’s National Media Digitization Construction in the Post-COVID-19 Era

**DOI:** 10.3389/fpsyg.2022.849097

**Published:** 2022-05-10

**Authors:** Yufei Tan, Xinlin She, Cun Zhou, Fangfei Wang

**Affiliations:** ^1^School of Culture and Communication, Capital University of Economics and Business, Beijing, China; ^2^School of Journalism and Communication, Hebei University, Baoding, China; ^3^Department of Journalism and Communication, Dalian University of Technology, Dalian, China

**Keywords:** self-determination theory, motivation, media users, national media, post-COVID-19 pandemic era

## Abstract

The sudden arrival of COVID-19 has had an enormous impact on the lives of people around the world, including significant psychological pressure and increased emotional needs. In China, research into user psychology and the motivations of commercial digital media has become more popular, but the national media should pay more attention to user psychology and perform more research on user motivations to improve the effectiveness of communication. We investigated people’s internal psychology and motivation for using national media digital platforms in China during the pandemic. We collected data through online questionnaires and analyzed the use of apps of CCTV and *The People’s Daily* by individual users during the pandemic and the psychological needs of national digital media users. In the first stage of our research, we selected national digital media app users through the WeChat platform. In the second stage, more active users were chosen by snowballing upon the original sample. We surveyed 210 participants and ultimately obtained 180 valid samples. We analyzed the data using used SPSS 23.0. The results showed that with the help of digital media platforms and diversified media technology, the Chinese national media not only met the needs of users for information acquisition, but also provided sufficient emotional mutual assistance and comfort to users through the network aggregation formed by digital scene communication.

## Introduction

The COVID-19 pandemic has significantly disrupted the world, and global systems’ capacity to respond to rapidly changing circumstances has been under enormous pressure (Remuzzi and Remuzzi, 2020). In the midst of this, social media has played a very important role in disseminating information about the pandemic. We have observed how digital social networks facilitate the exchange of information and emotions between people who are physically isolated (Díez García et al., 2020). A significant number of people have reported symptoms of stress, anxiety, and depression throughout the outbreak, and may have increased social media use as a result (Luo, 2021). At the same time, the spread of uncertainty in the social media environment has resulted in an enormous information upheaval, creating a transmission environment for the emotional contagion of anxiety and uncertainty in which people became highly insecure and constantly subjected to potential misinformation. The WHO calls the rapid spread of excessive information in the evolution of major pandemic outbreaks the “information pandemic” (WHO, 2020). The WHO believes that the large amount of unreliable information, such as rumors, spreads rapidly and internationally *via* the increasing popularity of mobile phones, social media, the Internet, and other communication technologies, resulting in the public lacking immediate and effective information, and subsequently delaying necessary pandemic intervention measures. Therefore, establishing a successful information management system to provide real information to meet the needs of the public may be able to effectively deal with such a crisis.

China’s national media, such as *The People’s Daily* and CCTV, are the most influential and widespread media outlets in China. They continue the tradition of news communication in the era of traditional media by diving deep into the informational requirements of pandemic prevention and control, addressing the psychological needs of the people, and effectively identifying and integrating information, in stark contrast to the fragmented nature of social media and the dissemination of uncertain information. People had stronger credibility-based attitudes toward mainstream media than toward alternative media (Xiao, 2021). However, China’s national media adopts innovations in media technology and trends in traditional media technology transformation, promoting both the digital integration of media with the help of new media technologies and new communication patterns. They do this in an attempt to alleviate the negative aspects of the pandemic for their audience with immersion and empathic communication, such as “micro-communication” and “slow live broadcasting.” The media has played a key role in stabilizing the mood of the people and helping them to readjust their social lives. The digital media at the provincial, municipal, and county levels also cooperate closely with each other and show remarkable effectiveness in transmitting information.

In the post-COVID-19 era, the public’s psychological healing, social reconstruction, and social development are closely related to the media ecology. At the same time, 5G, big data, cloud network integration, and edge computing are accelerating changes in communication patterns and structures. Under the support and drive of computer technology, internet technology, and digitalization technology, digital new media is developing rapidly in China (Wu, 2019). Currently, traditional media are accelerating the transformation of digitization, mobility, and intelligence.

From the perspective of media technology shaping human perception, scholars, such as Schmidt (1998), regard the media as the coupling of communication and cognition, as well as between media, communication, cognition, and culture, ultimately forming the “reality” identified by human beings (Sun, 2020). Thus, the transformation from traditional mass media to interpersonal communication is an important factor affecting the integration and development of digital media.

Social media has become an online platform to build disaster resilience among communities (Dufty, 2012). Specifically, social media is helpful in four aspects of disaster resilience: information gathering, information dissemination, collaborative problem-solving, and coping (Jurgens and Helsloot, 2018). When viewed at a macro level, real action has implications for the community’s community resilience in facing the COVID-19 pandemic. The conversation on Twitter during disaster enriches community-based resilience (Kalaloi et al., 2021). The versatility and goal-oriented nature of WeChat groups allow many of them to serve as emergent groups because they perform non-regular tasks and expand in size rapidly in a disaster setting. This dynamic growth lets group members gather otherwise inaccessible social capital. Those who have joined and engaged more in WeChat group activities have also received more aid from a diverse range of social connections (Chu and Yang, 2020). Some studies have shown that continued consumption of COVID-19-related media may aggravate psychological anxiety and pain in some individuals. COVID-19 reports have resulted in feelings of confusion, anxiety, and panic (Dong and Zheng, 2020; Holmes et al., 2020; Lăzăroiu et al., 2020; Rommer et al., 2020; Ullah and Amin, 2020). Based on the strong impact that the media may have on individuals, and considering the impact of the pandemic and the digital development of China’s national media, facing and meeting users’ motivation is therefore crucial. The value and feasibility of the development of media digital integration is based on the needs of the audience for various media content, therefore indicating that media users tend to choose a single supplier that can meet various related needs (Gambardella and Torrisi, 1998). In the post-COVID-19 pandemic era, the analysis of the use of state media by audiences should be a prominent component of the relevant research areas of national media construction.

The analysis of audience motivation in the integration and development of mainstream media and digital media uses self-determination theory (SDT). SDT (Deci and Ryan, 1980) focuses on autonomy and has recently expanded to include studies of awareness, integrative emotion regulation, life goals, the inner compass, needs crafting, revitalization, and self-talk, among other topics and processes relevant to understanding healthy agency (Deci and Ryan, 2000). As Koestner and Holding (2021) highlight, SDT is unique in its emphasis on this concept, suggesting that SDT’s emphasis on autonomy has particular salience in the era of COVID-19, in which voluntary compliance matters to public health.

## Materials and Methods

### Construction of the Measurement Model and Hypothesis of Media User’s Motivation

**Figure 1:**
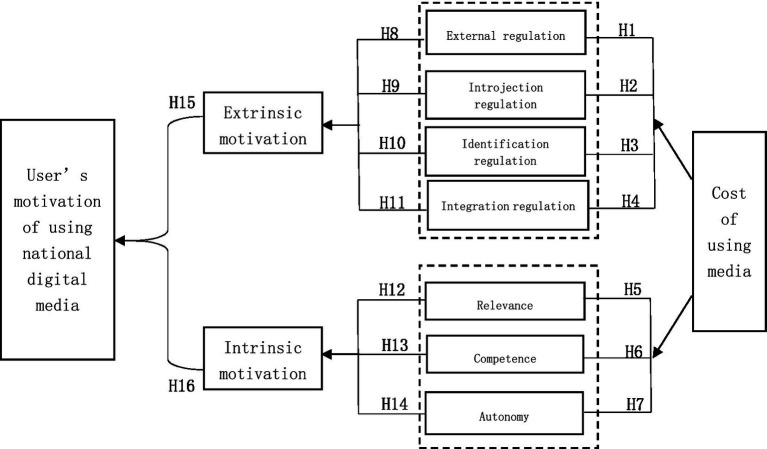
Subvariables of media user’s external and internal motivations.

Based on the relationship between extrinsic and intrinsic motivation of SDT (Deci and Ryan, 1980), this paper mainly discusses the dominant motivation of national digital media user behavior. As shown in Figure 1, the user’s external and internal motivations are divided into seven subvariables: external regulation, internal regulation, identity regulation, integration regulation, relevance, ability, and autonomy. Time cost has a positive impact on the classification results of the seven subdrivers. Among them, external supervision, internal supervision, identification supervision, and integrated supervision have a positive impact on the external drivers, while relevance, ability, and autonomy have a positive impact on the internal drivers, ultimately having a positive impact on the user’s motivation for using national digital media.

External regulation means that users receive additional rewards by meeting some external requirements, and these users are the least loyal in their use of national digital media applications. Integration regulation is the most internalized motivation type, meaning that individuals actively obtain information from and agree with the values and ethics transmitted by national digital media. As a result, these users are more receptive and loyal, which shows that people self-regulate their behavior, actively learn and master their own world, strive to internalize cultural norms, reflectively consider their own attitudes and values, and make informed choices (Deci and Ryan, 2000). An individual’s external behavior motivation dictates their opportunity to make their own decisions, resulting in higher levels of external motivation (Deci and Ryan, 2008).

The emotional tie of users to the country, to society, and to various groups is the specific manifestation of the internal motivation affecting users’ use of national digital media. Online interactions are positively associated with the general public’s psychological wellbeing (Yang et al., 2020). During the pandemic, users’ insightful comments on national digital media platforms are liked and forwarded by other users, and the information is shared by the community members through the national digital media apps, which can strengthen the sense of emotional belonging between users and the national digital media platform. Assumptions about the relationships of the variables are shown in Table 1.

**Table 1:** Research hypotheses of media user’s motivation.

H1: Use cost positively affects external adjustment
H2: Use cost positively affects input adjustment
H3: Positive impact of use cost on identity adjustment
H4: Use cost has a positive impact on integration and adjustment
H5: Use cost has a positive impact on the sense of belonging
H6: Use cost has a positive impact on competency
H7: Use cost has a positive impact on the sense of autonomy
H8: External regulation has a positive impact on external drivers
H9: External motivation has a positive impact on input regulation
H10: Positive influence of identity regulation on external motivation
H11: Positive impact of integration and regulation on external drivers
H12: A sense of belonging positively affects internal motivation
H13: Competency positively affects internal motivation
H15: External motivation positively affects user’s motivation
H16: Internal motivation positively affects user’s motivation

### Generation of a Multidimensional Measurement Scale for Media User’s Motivation

We randomly selected users to investigate their use of CCTV and daily apps from 20 January 2020 to 29 February 2020.

According to the characteristics of high efficiency, fragmentation, transience, interactivity, and authority of national digital media apps, the representative national digital media (CCTV and *The People’s Daily* apps) were selected, and a multidimensional measurement scale of relevant user motivations is proposed below. As shown in Table 2, according to SDT, the scale includes 2 primary indicators, 7 secondary indicators, and 13 measurement indicators. In addition, based on CCTV multidimensional measurement scale and the motivation of users of *The People’s Daily* app, the questionnaire of this study adopts the Likert method to quantify various indicators, represented by 1 = strongly disagree, 2 = disagree, 3 = uncertain, 4 = agree, and 5 = strongly agree (Table 3).

**Table 2:** Multidimensional measurement scale of user’s motivation of CCTV app and *The People’s Daily* app.

Primary index	Secondary index	Tertiary index	Measurement index	Number of topics
Extrinsic motivation	External regulation	Material reward	I use the CCTV and *People’s Daily* apps because there are bonuses.	2
		Other rewards	I use the CCTV and *People’s Daily* apps because my friends ask me so that they can get rewards.	
	Introjection regulation	Individual honor	I use the CCTV and *People’s Daily* apps because the number of users increased during the pandemic, so I have more chances to get more attention.	3
			I use the CCTV and *People’s Daily* apps because my friends, my family, and I can appear on the platform.	
		Time–cost	I use the CCTV and *People’s Daily* apps because the authoritative information they release can save me time.	
	Identification regulation	Social capital	I use the CCTV and *People’s Daily* apps to attract more listeners by publishing my views on the pandemic.	2
		Individual capital	I use the CCTV and *People’s Daily* apps because the national digital media has carried out online job fairs, such as the “national employment action” on the CCTV app.	
	Integration regulation	Individual trust	I use the CCTV and *People’s Daily* apps because I trust the goods they sell through their webcasts.	2
			I use the CCTV and *People’s Daily* apps because I trust their news content.	
Intrinsic motivation	Relatedness	Social interaction needs	I use the CCTV and *People’s Daily* apps because my friends discuss pandemic news from these apps.	2
		National destiny	I use the CCTV and *People’s Daily* apps because I am concerned about national actions, such as the construction process of the Huashan Mountain and Leishen Mountain hospitals.	
	Competence		I use the CCTV and *People’s Daily* apps because the content I publish on this platform can be recognized by many other audiences.	1
	Autonomy		I use the CCTV and *People’s Daily* apps because they let me participate in the discussion of national news.	1
Total				13

**Table 3:** Likert scale of user’s motivation of CCTV app and *The People’s Daily* app.

I use the CCTV and *People’s Daily* apps because there are bonuses
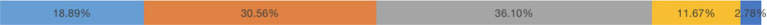
I use the CCTV and *People’s Daily* apps because my friends ask me so that they can get rewards
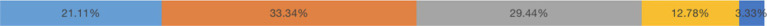
I use the CCTV and *People’s Daily* apps because the number of users increased during the pandemic, so I have more chances to get more attention
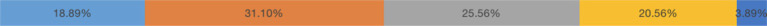
I use the CCTV and *People’s Daily* apps because my friends, my family, and I can appear on the platform.
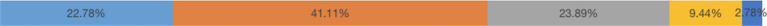
I use the CCTV and *People’s Daily* apps because the authoritative information they release can save me time.
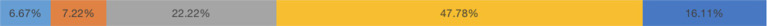
I use the CCTV and *People’s Daily* apps to attract more listeners by publishing my views on the pandemic.
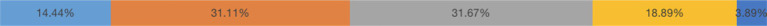
I use the CCTV and *People’s Daily* apps because the national digital media has carried out online job fairs, such as the “national employment action” on the CCTV app
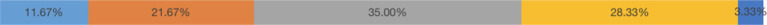
I use the CCTV and *People’s Daily* apps because I trust the goods they sell through their webcasts
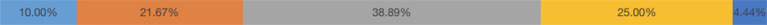
I use the CCTV and *People’s Daily* apps because I trust their news content
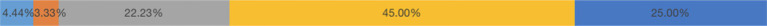
I use the CCTV and *People’s Daily* apps because my friends discuss pandemic news from these apps
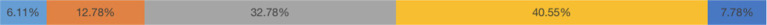
I use the CCTV and *People’s Daily* apps because I am concerned about national actions, such as the construction process of the Huashan Mountain and Leishen Mountain hospitals
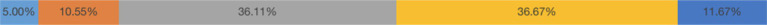
I use the CCTV and *People’s Daily* apps because the content I publish on this platform can be recognized by many other audiences
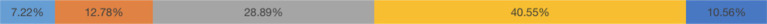
I use the CCTV and *People’s Daily* apps because they let me participate in the discussion of national news
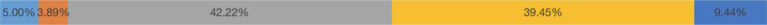

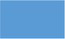
 Strongly disagree; 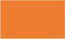 Disagree; 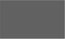 Uncertain; 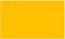 Agree; 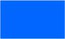 Strongly agree.

### Participants

The data were obtained *via* online questionnaires. We selected the apps of CCTV and *People’s Daily* users in the high incidence stage of the pandemic (from 20 January 2020 to 29 February 2020). In the first stage, the users of national digital media apps were randomly selected through the WeChat platform as the initial parent sample. In the second stage, more active users were chosen by snowballing the parent samples. We ultimately surveyed 210 individuals and obtained 180 valid samples. Half of the sample were female, while medical personnel accounted for approximately one-third of the sample. According to the grade of China’s pandemic prevention and control, there are 62 respondents in high-risk areas, 57 in medium-risk areas, and 61 in low-risk areas.

The results show that users in high-risk areas spent 9.62 h online every day, compared to 7.21 h in medium-risk areas and 6.16 h in low-risk areas. They spent approximately 2.88 h using national digital media applications, compared to 1.59 h in medium risk areas and 1.44 h in low-risk areas. Women spent 7.85 h online every day, compared to 5.8 h for men, and that they spent approximately 1.59 h using national digital media applications, compared to men’s usage of roughly 1.58 h. Medical personnel and non-medical personnel accounted for 31.1 and 68.9% of users, respectively, and medical personnel spent approximately 5.62 h online every day, while non-medical personnel spent approximately 6.90 h on average. Moreover, medical staff spent approximately 1.33 h on national digital media apps, and non-medical staff spent approximately 1.65 h on them.

## Results

### Construct Validity Analysis

We used SPSS 23.0 to analyze and process all data. Internal consistency was assessed using Cronbach’s α, structural validity was explored through exploratory factor analysis, and the correlation between the indexes of the above model was assessed by correlation analysis. As shown in Table 4, the chi-square statistical value of the KMO sample measure was 0.85, and the Bartlett sphere was 1396.53, where *p* < 0.001, indicating good validity.

**Table 4:** KMO and Bartlett’s test.

Kaiser–Meyer–Olkin Measure of Sampling Adequacy		0.85
Bartlett’s Test of Sphericity	Approx. Chi-square	1396.53
	df	91
	Sig.	< 0.001

### Reliability Analysis

Table 5 shows the reliability of 14 questions in the scale of from 180 questionnaires. Reliability was at 0.82, indicating good reliability.

**Table 5:** Reliability statistics.

Cronbach’s Alpha	No. of Items
0.823	14

Table 6 shows that only the correlation between identity adjustment and external drivers was significant, therefore supporting H3. It also supports the theory that users’ choice of national digital media platforms is often interest-centered and strongly influenced by personal purposes.

**Table 6:** Correlations (extrinsic motivation).

		External Regulation	Introjection Regulation	Identification Regulation	Integration Regulation	Total daily usage time of the CCTV and *The People’s Daily* apps
External Regulation	Pearson Correlation	1				
	Sig. (2-tailed)					
	N	180				
Introjection Regulation	Pearson Correlation	0.69^**^	1			
	Sig. (2-tailed)	0.001				
	N	180	180			
Identification Regulation	Pearson Correlation	0.60^**^	0.680^**^	1		
	Sig. (2-tailed)	0.001	0.001			
	N	180	180	180		
Integration Regulation	Pearson Correlation	0.32^**^	0.56^**^	0.56^**^	1	
	Sig. (2-tailed)	0.001	0.001	0.001		
	N	180	180	180	180	
Total daily usage time of CCTV video and People’s Daily app	Pearson Correlation	−0.15	0.03	0.15^**^	0.09	1
	Sig. (2-tailed)	0.84	0.65	0.04	0.19	
	*N*	180	180	180	180	180

** means more significant.

As in Table 6, H5 in Table 7 was similarly supported, suggesting that time-use cost is closely related to the sense of belonging. In the context of the pandemic, “contactless behavior” strengthens the audience’s online presence and permanent connection to the media, resulting in the “fear of missing out” phenomenon related to official pandemic-related information. This phenomenon also bears resemblance to the “dilemma of outsiders,” referring to the psychological state of individuals who are afraid of being excluded by the external world and fear that they will miss any changes. This was particularly a factor in the wake of the devastation provoked by the massive amount of COVID-19 deaths, often in the absence of loved ones. Among isolated people, trust guides people’s emotions. Trust is not only needed to gain access to knowledge, but it is also essential to becoming a socially situated person. It is internalized through social practices like support and cooperation (Simone and Claudia, 2021). In the wake of the social changes and turbulence brought about by the pandemic, the need for individuals to obtain real information and make emotional connections with the external world is particularly urgent (Zhou, 2020).

**Table 7:** Correlations (intrinsic motivation).

		Competence	Autonomy	Relatedness	Total daily usage time of CCTV and *The People’s Daily* apps
Competence	Pearson Correlation	1			
	Sig. (2-tailed)				
	N	180			
Autonomy	Pearson Correlation	0.50^**^	1		
	Sig. (2-tailed)	0.001			
	N	180	180		
Relatedness	Pearson Correlation	0.46^**^	0.51^**^	1	
	Sig. (2-tailed)	0.001	0.001		
	N	180	180	180	
Total daily usage time of CCTV video and People’s Daily app	Pearson Correlation	0.118	0.099	0.175^*^	1
	Sig. (2-tailed)	0.114	0.188	0.018	
	*N*	180	180	180	180

* means significant;

** means more significant.

As shown in Table 8, the assumptions H8, H9, H10, H11, H12, H13, h14, H15, and H16 made by the above model reached significant levels (*p* < 0.01).

**Table 8:** Bivariate correlations (primary and secondary indicators).

		Extrinsic motivation	Intrinsic motivation	User’s motivation
External regulation	Pearson Correlation	0.80^**^	0.15^*^	0.67^**^
	Sig. (2-tailed)	0.001	0.03	0.001
	N	180	180	180
Introjection regulation	Pearson Correlation	0.90^**^	0.46^**^	0.86^**^
	Sig. (2-tailed)	0.001	0.001	0.001
	N	180	180	180
Identification regulation	Pearson Correlation	0.85^**^	0.44^**^	0.81^**^
	Sig. (2-tailed)	0.001	0.001	0.001
	N	180	180	180
Integration regulation	Pearson Correlation	0.71^**^	0.66^**^	0.78^**^
	Sig. (2-tailed)	0.001	0.001	0.001
	N	180	180	180
Relatedness	Pearson Correlation	0.38^**^	0.88^**^	0.61^**^
	Sig. (2-tailed)	0.001	0.001	0.001
	N	180	180	180
Competence	Pearson Correlation	0.50^**^	0.76^**^	0.66^**^
	Sig. (2-tailed)	0.001	0.001	0.001
	N	180	180	180
Autonomy	Pearson Correlation	0.38^**^	0.76^**^	0.56^**^
	Sig. (2-tailed)	0.001	0.001	0.001
	N	180	180	180
Extrinsic motivation	Pearson Correlation	1	0.50^**^	0.95^**^
	Sig. (2-tailed)		0.001	0.001
	N	180	180	180
Intrinsic motivation	Pearson Correlation	0.50^**^	1	0.74^**^
	Sig. (2-tailed)	0.001		0.001
	*N*	180	180	180

* means significant;

** means more significant.

## Discussion

As a result of the pandemic, non-contact behavior suddenly forced everyone to coordinate and deal with environmental, physical, psychological, and emotional discomfort and confusion on a large scale.

Media integration is not only the integration of media technology, but also the integration of collective human sensory experience and the resulting integration of emotions and thoughts. This is particularly true when people face an environment that they cannot experience personally, resulting in the closer integration of people and technology in the post-COVID-19 pandemic era.

Media integration also refers to the transformation of communication relations. The initiative, selectivity, enthusiasm, and participation of the audience play a great role, and the relationship between the media and the audience can be significantly transformed.

The national digital media should pay attention to the internal needs of people’s psychology, provide authoritative emotional support for their audience’s pressures and fears through emotional narration, and potentially alleviate negative thoughts through effective empathy.

In the post-COVID-19 pandemic era, the construction of national digital media should meet the audience’s needs to participate in the world, and build a media environment to adapt to the new ecological environment under the altered social structure.

The role of emotional communication has been extremely prominent during the pandemic. In the post-COVID-19 era, the emotional function of national digital media has gradually shifted to realizing social emotional resonance, recognition, and motivation, and promoting cognition, joint force, and investment in social co-construction. Therefore, the national media has begun to bear responsibility for the restoration and reconstruction of social order and construction, actively mobilizing and forging pathways toward emotional relief, and unifying individual emotions into a powerful force to promote the transformation of actions in the post-pandemic era.

## Conclusion and Implications

Based on self-determination theory (SDT), this study analyzed the use of CCTV’s and *The People’s Daily’s* apps by individual users during the pandemic, as well as the psychological needs of national digital media users, and found the following. First, there is a correlation between pandemic risk and people’s media usage time. People in higher risk areas spent more time online. Second, users’ choice of national digital media applications is influenced by personal extrinsic motivation. Users’ choice of national digital media platforms is often interest-centered and strongly influenced by personal purposes. Third, users’ choice of national digital media applications is also influenced by personal intrinsic motivation. Due to the lack of sense of belonging caused by “contactless behavior” in the pandemic, the need for individuals to obtain real information and make emotional connections with the external world is particularly urgent.

In the post-COVID-19 era, emotion and social psychology are the important influencing variables of media communication. The internal needs of the media audience, therefore, should be considered and gradually become the driving force of the transformation of the production and communication modes of China’s national media. In the digital media environment, China’s national media outlets should gradually adopt a method of emotional communication to innovate and transform production mechanisms and communication modes.

## Data Availability Statement

The raw data supporting the conclusions of this article will be made available by the authors, without undue reservation.

## Author Contributions

YT: thesis design, drafted the paper, made important modifications to the paper, and approved the final paper to be published. XS: data collection and analysis, drafted the paper, and data verification. FW: data analysis, drafted the paper, and made important modifications to the paper. CZ: reference collection and data analysis. All authors contributed to the article and approved the submitted version.

## Conflict of Interest

The authors declare that the research was conducted in the absence of any commercial or financial relationships that could be construed as a potential conflict of interest.

## Publisher’s Note

All claims expressed in this article are solely those of the authors and do not necessarily represent those of their affiliated organizations, or those of the publisher, the editors and the reviewers. Any product that may be evaluated in this article, or claim that may be made by its manufacturer, is not guaranteed or endorsed by the publisher.
